# Effects of Low-Dose Recombinant Human Brain Natriuretic Peptide on Anterior Myocardial Infarction Complicated by Cardiogenic Shock

**DOI:** 10.21470/1678-9741-2016-0007

**Published:** 2017

**Authors:** Yesheng Pan, ZhiGang Lu, Jingyu Hang, Shixin Ma, Jian Ma, Meng Wei

**Affiliations:** 1 Heart Center, Shanghai Jiao Tong University Affiliated Sixth People's Hospital, Shanghai, P.R. China.

**Keywords:** Myocardial Infarction. Shock, Cardiogenic. Natriuretic Peptide, Brain

## Abstract

**Introduction:**

The mortality due to cardiogenic shock complicating acute myocardial infarction (AMI) is high even in patients with early revascularization. Infusion of low dose recombinant human brain natriuretic peptide (rhBNP) at the time of AMI is well tolerated and could improve cardiac function.

**Objective:**

The objective of this study was to evaluate the hemodynamic effects of rhBNP in AMI patients revascularized by emergency percutaneous coronary intervention (PCI) who developed cardiogenic shock.

**Methods:**

A total of 48 patients with acute ST segment elevation myocardial infarction (STEMI) complicated by cardiogenic shock and whose hemodynamic status was improved following emergency PCI were enrolled. Patients were randomly assigned to rhBNP (n=25) and control (n=23) groups. In addition to standard therapy, study group individuals received rhBNP by continuous infusion at 0.005 µg kg^−1^ min^−1^ for 72 hours.

**Results:**

Baseline characteristics, medications, and peak of cardiac troponin I (cTnI) were similar between both groups. rhBNP treatment resulted in consistently improved pulmonary capillary wedge pressure (PCWP) compared to the control group. Respectively, 7 and 9 patients died in experimental and control groups. No drug-related serious adverse events occurred in either group.

**Conclusion:**

When added to standard care in stable patients with cardiogenic shock complicating anterior STEMI, low dose rhBNP improves PCWP and is well tolerated.

**Table:** 

Abbreviations, acronyms & symbols				
ACEI	= Angiotensin converting enzyme inhibitors		LMWH	= Low-molecular-weight heparin
AMI	= Acute myocardial infarction		LVADs	= Left ventricular assist devices
ANOVA	= Analysis of variance		LVEF	= Left ventricular ejection fraction
ANP	= Atrial natriuretic peptide		PCI	= Percutaneous coronary intervention
ARB	= Angiotensin receptor blockers		MSOF	= Multiple systemic organ failure
ASCEND-HF	= Acute Study of Clinical Effectiveness of Nesiritide		NT-proBNP	= N-terminal brain natriuretic peptide
	and Decompensated Heart Failure		PCWP	= Pulmonary capillary wedge pressure
BNP	= B type natriuretic peptide		RAP	= Right atrial pressure
CABG	= Coronary artery bypass graft surgery		rhBNP	= Recombinant human brain natriuretic peptide
cGMP	= Cyclic guanosine monophosphate		SBP	= Systolic blood pressure
CI	= Cardiac index		SOAP	= Sepsis occurrence in acutely ill patients
cTnI	= Cardiac troponin I		SPSS	= Statistical Package for the Social Sciences
ECG	= Eletrocardiograma		STEMI	= ST segment elevation myocardial infarction
eGFR	= Estimate glomerular filtration rate		VMAC	= Vasodilation in the Management of Acute
FDA	= U. S. Food and Drug Administration			Congestive Heart Failure
IABP	= Intra-aortic balloon pump			

## INTRODUCTION

Cardiogenic shock complicates 6-10% of all ST segment elevation myocardial infarction (STEMI) cases and remains a leading cause of death, with hospital mortality rates approaching 50%^[1]^. Emergency revascularization with percutaneous coronary intervention (PCI) or coronary artery bypass graft surgery (CABG) has been shown to improve long-term survival of these patients^[2-4]^. Despite this progress, cardiogenic shock continues to be associated with a high short-term mortality rate^[5]^. Vasopressors and inotropes are used to improve the hemodynamic status, but the information about comparative effective outcomes is limited; indeed, some of them could induce decreased survival rate that may be associated with the deleterious cellular effects of these drugs^[6,7]^.

B type natriuretic peptide (BNP), released due to altered chamber loading and myocyte stretch, has been detected in acute myocardial infarction (AMI)^[8,9]^. BNP can act via the natriuretic peptide receptor A/guanylyl cyclase/cyclic guanosine monophosphate (cGMP) signaling pathway, maintaining cardio-renal homeostasis through diuresis, natriuresis, vasodilation, and inhibition of aldosterone synthesis and renin secretion under physiological and pathological conditions^[10]^. Interestingly, exogenous BNP infusion may result in coronary vasodilatation with reduced myocardial oxygen consumption^[11]^, enhanced myocardial relaxation^[12]^, suppressed synthesis and release of aldosterone^[13]^, induced vascular regeneration^[14]^, delayed adrenergic activation and inhibited cardiac fibroblast collagen synthesis^[15-17]^.

rhBNP (Recombinant human brain natriuretic peptide) was approved by the U. S. Food and Drug Administration (FDA) for the management of acute decompensated heart failure in 2001. Recently, a small study showed that rhBNP, given soon after AMI, induces a trend towards favorable ventricular remodeling, but blood pressure was lower with rhBNP over the first 8-12 hours of infusion without clinically significant hypotension^[18]^. Low-dose rhBNP administration could avoid hypotension in patients with cardio-renal disease. Indeed, Brunner-La Rocca et al.^[15]^ have reported that in humans withheart failure, low-dose rhBNP suppresses the adrenergic outflow without hypotension. Chen et al.^[13]^ reported that 72 hours of infusion of low-dose rhBNP at the time of anterior AMI is well tolerated, and may suppress aldosterone and preserve ventricular function and structure in patients with anterior AMI.

There are currently no reports on low-dose rhBNP administration in patients with cardiogenic shock complicating STEMI after successful early revascularization. We proposed a pilot study to evaluate the safety and efficacy of low dose rhBNP in the setting of STEMI complicated with cardiogenic shock. This study was designed to evaluate the hemodynamic, neuro-hormonal, and renal effects of rhBNP given as a 72-hour infusion to AMI patients revascularized by emergency PCI who developed cardiogenic shock, but were stable after appropriate medical intervention.

## METHODS

### Patients

The study group included patients admitted to Shanghai 6^th^ People's Hospital, China, with cardiogenic shock complicating a first anterior AMI from March 2009 to March 2013. Patients were eligible for the trial if they presented with: (1) anterior STEMI complicated by cardiogenic shock; (2) successful early revascularization performed with stable hemodynamic status in the 1^st^ hour post-intervention. Anterior STEMI was diagnosed by the following criteria: prolonged chest pain >30 minutes; positive troponin I; ST-segment elevation 2 mv in two or more adjacent anterior precordial leads. A patient was considered to be in cardiogenic shock if: (1) systolic blood pressure was less than 90 mmHg for more than 30 minutes or catecholamine infusion was required to maintain a systolic pressure above 90 mmHg; (2) clinical signs of pulmonary congestion were present; or (3) there was evidence of impaired end organ perfusion. The diagnosis of impaired end organ perfusion required at least one of the following: altered mental status; cold, clammy skin and extremities; oliguria with urine output of less than 30 ml per hour; serum lactate level higher than 2.0 mmol per liter. All patients got successful revascularization (emergency PCI, TIMI grade 3 flow) and intra-aortic balloon pump (IABP) support within 24h of onset of chest pain, were enrolled, and sent to Coronary Care Unit for hemodynamic monitoring.

Exclusion criteria were: SBP < 90 mmHg within first hour post-intervention although 12 mg/kg × min dopamine and 1:1 IABP supporting; pulmonary capillary wedge pressure (PCWP) < 18 mmHg; acute inferior, posterior and right ventricle myocardial infarction; previous history of myocardial infarction; previous eletrocardiograma (ECG) suggesting an old myocardial infarction; previous history of chronic heart failure or decreased left ventricular ejection fraction (LVEF); hypertrophic cardiomyopathy; hemodynamically significant mitral regurgitation; other hemodynamically significant valvular or congenital heart diseases; estimate glomerular filtration rate (eGFR) < 15 mL/min per 1.73 m^2^; previous known intolerance history to rhBNP.

This was a randomized (sealed enveloped), open-label, parallel group study. Patients were assigned to rhBNP or control group at a 1:1 ratio. The primary endpoints were absolute changes in PCWP from baseline to 72 hours after randomization compared to the control group. Secondary endpoints included comparisons between rhBNP and control groups of the following hemodynamic and clinical effects: cardiac index, in hospital mortality, 72 hours urine output, and safety profile (renal function, hypotension). The trial protocol was approved by the ethics committee of Shanghai 6^th^ People's Hospital. Documented informed consent was obtained from each patient or his/her guardian.

### Protocol

Each patient was monitored hemodynamically using a pulmonary artery Swan-Ganz thermodilution catheter (bedside chest X-ray was done to check its position). Arterial blood pressure was measured with IABP. Baseline heart rate, blood pressure, right atrial pressure (RAP), PCWP and cardiac index (CI) were recorded three times within a 5 minutes period using the bedside patient monitor. Measurements were carried out by another research team member blinded to treatment allocation. Additional measurements were performed 15 min and then 3, 24, 48 and 72h after starting drug administration. Echocardiography was performed immediately after operation and read in random order with no patient identifiers.

All the patients were given dopamine (3-12 mg/kg × min) after diagnosis of cardiogenic shock, at dosage adjusted to achieve a satisfactory blood pressure (SBP < 90 mmHg). Patients with stable blood pressure (SBP < 90 mmHg) within 1 hour post-intervention were enrolled and randomized. In the rhBNP group, rhBNP treatment was initiated after randomization for 72 hours with a continuous infusion of 0.005 mg kg^-1^min^-1^ without a loading dose. The infusion dosage was decreased to 0.003 mg kg^-1^min^-1^ if SBP < 85 mmHg more than 20 minutes despite dopamine titration. If symptomatic hypotension or systolic blood pressure < 75 mmHg occurred, rhBNP infusion was interrupted for 30-60 minutes and the dosage of dopamine increased. rhBNP treatment would restart at 0.003 mg kg^-1^min^-1^ when the dose-limiting event had resolved. Infusion of rhBNP was discontinued for recurrent symptomatic hypotension; sustained hypotension despite rhBNP dosage decrease to 0.003 mg kg^-1^min^-1^ or dopamine dosage increase to more than 12 mg kg^-1^min^-1^.

In addition to standard medical treatment, dobutamine and norepinephrine were permitted for intractable hypotension and pump failure based on investigator's clinical judgment. Statin, aspirin and clopidogrel were given before emergency PCI. IIb/IIIa receptor antagonist (Tirofiban) was administered during the procedure and next 24 hours according to the physician's judgment. Enoxaparin was given from 6 to 72 hours after emergency PCI. Low dose of angiotensin converting enzyme inhibitors (ACEI) and angiotensin receptor blockers (ARB) as well as beta-blocker therapy were started 24h after randomization if patients tolerated. Blood for measurement of N-terminal brain natriuretic peptide (NT-proBNP), BNP, cGMP and creatinine levels was drawn before the initiation of IV rhBNP, then at 72 hours and 1 week after randomization.

### Statistical Analysis

We postulated an expected change of PCWP as -3.8 mmHg from Vasodilation in the Management of Acute Congestive Heart Failure (VMAC) study^[19]^. We calculated that a sample size of 21 patients would be needed in each group for a statistical power of 80% at significance level of 0.05 (with a two-sided t-test).

Data were statistically processed and analyzed using the Statistical Package for the Social Sciences (SPSS) 16.0 software package. Descriptive statistics were presented as mean±standard deviation for continuous data and as percentages for categorical data. Analyses of continuous data were performed by two-sample t-test and categorical data by Chi-Square test. One way analysis of variance (ANOVA) was used to evaluate the differences among groups after treatment. The changes in renal function from baseline to after drug administration were analyzed by paired t-test. *P* <0.05 was considered statistically significant.

## RESULTS

A total of 65 patients were screened, of whom 17 were excluded because of hypotension (n=8), death before randomization (n=3), PCWP < 18 mmHg (n=3), eGFR < 15 mL/min per 1.73 m^2^ (n=2) or significant mitral valve regurgitation (n=1).

Table 1 summarizes the baseline characteristics of patients, which were similar between both groups. Indeed, peak cTnI, time to PCI, PCWP, CI and LVEF were comparable between the two groups at baseline.

**Table 1:** Baseline characteristics of the patients.

Characteristics	rhBNP (n=25)	Control (n=23)
Age - years	64.9±12.6	64.1±10.8
Male sex - no. (%)	18 (72)	17 (74)
Body mass index	23.5±2.8	24.2±2.7
**Cardiovascular risk factors - no. (%)**		
Smoking	14 (56%)	12 (52%)
Hypertension	12 (48%)	15 (65%)
Diabetes mellitus	9 (36%)	7 (30%)
Time to PCI (h)	12.7±5.0	13.1±5.1
**Infarct-related artery - no. (%)**		
Left main	4 (16%)	4 (17%)
Left anterior descending	21 (84%)	18 (78%)
Left circumflex	__	1 (4%)
Multivessel disease - no. (%)	2.0±0.9	1.8±0.8
Peak cTnI (mg/L)	99.7±55.1	93.9±46.7
Ejection fraction (%)	38.6±8.0	39.6±7.2
eGFR (mL/min - 1.73m^2^)	65.4±28.6	67.2±33.6
Cardiac index (L/min - m^2^)	1.7±0.2	1.7±0.2
PCWP (mmHg)	27.2±4.1	27.0±4.2

Data are presented as mean value ± SD Body-mass index is the weight in kilograms divided by the square of the height in meters. eGFR = estimate glomerular filtration rate; PCI = percutaneous coronary intervention; PCWP = pulmonary capillary wedge pressure; rhBNP = recombinant human brain natriuretic peptide

Table 2 shows the clinical management in the hospital. All the patients underwent successful PCI and IABP support. Rates of noninvasive positive pressure ventilation were similar for both of them. Standard medical therapy was used unless contraindicated. ACEI/ARB and beta-blockers were used if tolerated and titrated gradually. Medical treatments in both groups were similar; no significant differences were observed in average dopamine dosage as well as dobutamine and epinephrine use between both groups.

**Table 2:** Clinical management in hospital.

Management	rhBNP (n=25)	Control (n=23)
Emergency PCI - no. (%)	25 (100)	23 (100)
Stenting	25 (100)	23 (100)
Emergency CABG - no. (%)	__	__
Intra-aortic balloon pump - no. (%)	25 (100)	23 (100)
Left ventricular assist device - no. (%)	__	__
Noninvasive positive pressure ventilation - no. (%)	12 (48)	12 (52)
**Medication in hospital**		
rhBNP administration - hours	60.4	__
**Dopamine- average dosage (mg/kg×min)**		
Mean value ± SD	7.5±5.2	8.7±5.5
Dobutamine - no. (%)	2 (8)	3 (13)
Norepineprine - no. (%)	3 (12)	2 (9)
Diuretic - no. (%)	22 (88)	21 (91)
Beta-blocker - no. (%)	20 (80)	16 (70)
ACE inhibitor - no. (%)	11 (44)	12 (52)
ARB - no. (%)	3 (12)	4 (17)
Statins - no. (%)	22 (88)	21 (91)
Clopidogrel - no. (%)	25 (100)	23 (100)
Aspirine - no. (%)	23 (92)	20 (87)
IIb/IIIa receptor antagonist - no. (%)	10 (40)	11 (48)
LMWH - no. (%)	25 (100)	22 (96)

ACE = angiotensin converting enzyme; ARB = angiotensin receptor blocker; CABG = coronary-artery bypass grafting; LMWH = lowmolecular- weight heparin; PCI = percutaneous coronary intervention

A total of 16 patients died in hospital. In the rhBNP group, 5 patients died within 72h of randomization [pump failure (n=4); multiple systemic organ failure - MSOF - (n=1)], and 1 patient died at each day 4 (cardiac rupture) and 10 (pump failure) after randomization. In the control group, 8 patients died within 72h after randomization [pump failure (n=5); MSOF (n=2); ventricular fibrillation (n=1) and 1 died at day 6 (pump failure). These findings indicated that there was no significant difference in in-hospital mortality between the two groups (28.0 vs. 39.1%; P=0.305). No re-infarctions or cerebrovascular accidents were observed in either group.

Hemodynamic characteristics at 3 and 72h after randomization were analyzed in live patients (Table 3). After rhBNP infusion, PCWP and RAP were significantly decreased compared to the control group; cardiac index showed increasing trend in the rhBNP group, but the differences were not statistically significant. No statistically significant differences were obtained for heart rate, SBP and mean blood pressure between both groups. The dose of rhBNP was reduced to 0.003 mg kg^-1^min^-1^ in 3 patients because of asymptomatic hypotension. In 2 patients of the study group, rhBNP infusion was discontinued for symptomatic hypotension, which resolved with no clinical consequences after infusion was stopped. The 72h urine volume after randomization showed no statistically significant difference between these two groups (rhBNP group, 2293 ml/d; control group, 2053 ml/d; P=0.11).

**Table 3:** Changes of hemodynamic parameters (compare to baseline).

Hemodynamic parameters	rhBNP (n=20)	Control (n=15)	*P* value
HR (bpm) baseline	96.7±11.3	99.1±15.8	0.601
HR (bpm) 72h	-14.0±8.8	-11.1±9.9	0.370
SBP (mmHg) baseline	105.8±11.6	102.7 ± 9.4	0.399
SBP (mmHg) 72h	-1.9±11.8	+2.0±12.1	0.346
MBP (mmHg) baseline	78.1±6.0	78.8±5.6	0.732
MBP (mmHg) 72h	-0.9±7.1	+0.5±8.1	0.591
RAP (mmHg) baseline	15.0±1.7	14.6±1.6	0.482
RAP (mmHg) 3h	-2.2±0.6	-1.5±1.1	0.015
RAP (mmHg) 72h	-4.9±1.6	-3.4±0.8	0.002
PCWP (mmHg) baseline	26.1±3.8	25.3±4.1	0.541
PCWP (mmHg) 3h	-5.5±2.6	-2.1±3.4	0.002
PCWP (mmHg) 72h	-9.3±3.6	-5.3±3.1	0.002
CI (L/min × m^2^) baseline	1.7±0.1	1.7±0.1	0.760
CI (L/min × m^2^) 3h	+0.1±0.1	0.0±0.1	0.122
CI (L/min × m^2^) 72h	+0.4±0.1	+0.3±0.2	0.079

Data are presented as mean value ± SD. CI = cardiac index; HR = heart rate; MBP = medium blood pressure; PCWP = pulmonary capillary wedge pressure; RAP = right atrium pressure; SBP = systolic blood pressure

As shown in Table 4, plasma BNP levels increased from baseline after rhBNP infusion, and decreased when the infusion was stopped. cGMP, a secondary messenger of BNP, increased in the rhBNP group compared to control patients. NT-proBNP and eGFR levels were not statistically different between the two groups. In addition, no statistically significant difference was observed in eGFR after rhBNP infusion (68.3±25.2 vs. 64.8±19.7 P=0.401).

**Table 4:** Changes of biomarkers and renal function.

Biomarkers	rhBNP (n=19)	Control (n=14)	*P* value
NT-proBNP (pg/ml) baseline	6097.5±3932.7	5315.1±5657.1	0.642
NT-proBNP (pg/ml) 72h after randomization	3820.0±3446.1	4605.2±5063.1	0.599
NT-proBNP (pg/ml) 1 week after randomization	2951.4±2122.8	3640.6±3256.1	0.467
BNP (pg/ml) baseline	1186.2±738.1	1113.1±1089.4	0.820
BNP (pg/ml) 72h after randomization	1603.4±672.6	925.4±1090.2	0.035
BNP (pg/ml) 1 week after randomization	609.0±464.0	670.0±711.2	0.767
cGMP (pmol/ml) baseline	5.0±1.0	4.8±1.3	0.587
cGMP (pmol/ml) 72h after randomization	6.2±1.3	4.1±0.8	<0.001
eGFR (mL/min×1.73 m^2^) baseline	68.3±25.2	81.4±34.4	0.216
eGFR (mL/min×1.73 m^2^) 72h after randomization	62.3±23.3	68.1±28.1	0.521
eGFR (mL/min×1.73 m^2^) 1 week after randomization	64.8±19.6	69.4±24.5	0.558

Data are presented as mean value ± SD (from patients alive on day 7). BNP = brain natriuretic peptide; cGMP = cyclic guanosine monophosphate; eGFR = estimate glomerular filtration rate; NT proBNP = N-terminal brain natriuretic peptide

## DISCUSSION

Animal and clinical studies have shown that BNP infusion limits infarct size, improves cardiac remodeling and protects cardiomyocytes^[15,17,20-22]^. Recently, a large multicenter clinical trial using atrial natriuretic peptide (ANP) at the time of myocardial reperfusion with AMI was reported that ANP (carparitide) could reduce infarct size, improve ejection fraction and decrease the rate of new-onset heart failure^[23]^. Meta-analysis suggested that ANP/BNP infusion might be effective in protecting left ventricular function in patients with AMI^[24]^. Thus natriuretic peptides (ANP and BNP) seem to be logical adjuncts to standard care for the treatment of AMI. Additionally, BNP has proven effects on acute decompensated heart failure, and should not decrease blood pressure at low dose^[13,15]^. Therefore, we hypothesized that BNP would be safe and effective for the treatment of STEMI patients with cardiogenic shock. In this study, 48 patients with cardiogenic shock complicating anterior STEMI, successful emergency PCI, and stable hemodynamic status post intervention were enrolled. The major findings of this study are: (1) in comparison with controls, administration of low-dose rhBNP for 72 hours is associated with PCWP reduction; (2) low dose rhBNP treatment is unlikely to be limited by hypotension in this clinical setting; (3) low dose rhBNP does not significantly affect the renal function. Patients with STEMI complicated by cardiogenic shock can benefit from the hemodynamic effects of rhBNP. On the other hand, the long-term benefits of remodeling inhibition and cardio-protection need to be confirmed by further studies. Mortality in this study was lower than what reported for the SHOCK trial^[3]^. In this work, patients who remained unstable after emergency PCI were excluded,which may explain the discrepancy.

In the American College of Cardiology/American Heart Association and European Society of Cardiology guidelines for STEMI, emergency revascularization by PCI or CABG for the cardiogenic shock complicating AMI is given a class IB recommendation^[25-26]^. In our hospital, emergency PCI is the first choice for these patients. IABP was the most widely used form of mechanical hemodynamic support in this clinical setting at the time of the present trial. In the current U.S. and European guidelines, IABP in the treatment of cardiogenic shock is still given IIa and IIb recommendations, respectively, although recent clinical trials showed IABP use does not significantly reduce the 30-day mortality rate in these patients^[25-28]^. Mechanical left ventricular assist devices (LVADs) have been used in cardiogenic shock patients not responding to standard therapy, but evidence regarding their benefits is limited. In this study, all patients were submitted to emergency PCI and IABP support, and no LVADs were used. The Sepsis Occurrence in Acutely Ill Patients (SOAP) II study was published during the early phase of this trial, with a subgroup analysis showing that dopamine, as compared with norepinephrine, was associated with increased mortality rate at 28 days in patients with cardiogenic shock^[29]^. However, dopamine is still given a IIa recommendation for AMI patients with CS in the current European Society of Cardiology STEMI guideline^[26]^. Here, norepinephrine and dobutamine were permitted for intractable hypotension and pump failure. All study patients were treated by the same medical team, to limit biases.

The effect of rhBNP on renal function is controversial. A meta-analysis showed that standard rhBNP dose (0.01 mg kg^-1^min^-1^) might be detrimental to renal function in patients with acute decompensated heart failure^[30]^; meanwhile, the multicenter randomized control trial Acute Study of Clinical Effectiveness of Nesiritide and Decompensated Heart Failure (ASCEND-HF) confirmed its renal safety^[31]^. Recently, a small study reported that low-dose rhBNP improves renal function in heart failure patients following AMI^[32]^. We demonstrated herein that renal function before and after treatment was similar between both groups.

BNP is a biologically active hormone affecting diuresis and natriuresis. However, increased urine output with physiological and common pharmacological doses of rhBNP has been proven only in normal individuals. The ASCEND-HF trial^[33]^ showed no evidence that nesiritide increases urine output in patients with acute decompensated heart failure. Here, we found no evidence that rhBNP increases urine output in the cardiogenic shock patients assessed.

Some small scale clinical trials suggested rhBNP infusion might protect left ventricular function in patients with AMI, but there is no randomized clinical trial which with large sample on these patients^[24]^. Based on our results, further clinical trial could be planned on rhBNP treatment for AMI patients with or without cardiogenic shock.

## CONCLUSION

When added to standard care in stable patients with cardiogenic shock complicating anterior STEMI, low dose of rhBNP improves PCWP and is well tolerated.

## LIMITATION

This was a small open control randomized pilot study. There was no clinical data for PCWP changes after 72h infusion of low dose rhBNP, so we postulated an expected PCWP change of -3.8 mmHg as shown in the VMAC study, which was designed with 3 hours rhBNP infusion at 0.01 mg kg^-1^min^-1^ after randomization. Further adequately powered trials are needed to test the short and long-term benefits of rhBNP infusion in this clinical setting.

**Table:** 

Authors' roles & responsibilities	
YP	Conception and design study; realization of operations
	and/or trials; analysis and/or data interpretation;
	statistical analysis; manuscript redaction or critical review of its content; final manuscript approval
ZGL	Final manuscript approval
JH	Manuscript redaction or critical review of its content; final manuscript approval
SM	Realization of operations and/or trials; final manuscript approval
JM	Statistical analysis; final manuscript approval
MW	Final manuscript approval
